# Formulation of Transfer Curves for Reversal Loadings Based on Soil–Concrete Interface Tests and Flat Dilatometer Soundings

**DOI:** 10.3390/ma18163798

**Published:** 2025-08-13

**Authors:** Kamila Mikina, Jakub Konkol

**Affiliations:** Faculty of Civil and Environmental Engineering, Department of Geotechnical and Hydraulic Engineering, Gdańsk University of Technology (GUT), 11/12 Gabriela Narutowicza Street, 80-233 Gdańsk, Poland; kamila.mikina@pg.edu.pl

**Keywords:** Controlled Modulus Columns (CMC), Flat Dilatometer Test (DMT), organic soil, pile–soil interaction, skin friction, soil–concrete interface, soft soils, transfer curves

## Abstract

This study introduces a novel method for evaluating pile–soil interaction based solely on Dilatometer Test (DMT) results, enhancing and extending the established approach originally developed using Menard Pressuremeter Test (PMT) data. Currently, transfer functions utilizing DMT sounding results are in the early stages of development. Presented research fills the gap in DMT-based methods for pile design by introducing transfer functions for reversal loadings to calculate the unit shaft friction of screw displacement piles in Controlled Modulus Columns (CMC) technology. The proposed method utilizes DMT-derived soil parameters, offering a practical and accurate alternative to PMT-based models. Testing research fields were located in the Vistula Marshlands, Northern Poland. Site characterization consisted of piezocone (CPTU) and DMT soundings to characterize the soil profile and estimate soil parameters relevant for pile design. CMCs were installed and statically load tested under various loading schemes. Laboratory direct shear tests on smooth and rough soil-concrete interfaces were performed in both forward and backward directions (reversal loading) to simulate pile loading conditions. Results demonstrate improved adaptability of DMT-based transfer curves to local soil conditions and provide a reliable framework for predicting pile performance in soft soils. Proposed DMT-model returns similar ultimate bearing capacities of the pile to CPT 2012 method for first loading, simultaneously offering better agreement for reversal loading, a situation not accounted for in CPTU 2012 or most other CPT-based methods.

## 1. Introduction

Two general approaches can be distinguished in pile bearing capacity calculations: indirect methods––based on soil strength properties determined in lab or from field investigation tests––and direct methods––based solely on registered parameters from field soundings. In direct pile design, soundings results are used to estimate both base and shaft resistance, without the need of determining additional soil parameters. Most field methods utilize data from static cone penetration tests (CPT or CPTu) [1,2,3,4,5,6], due to the data sampling frequency and relative ease of performing such tests. Moreover, a CPT may be considered a mini-pile, where cone tip resistance corresponds to base resistance, and the sleeve friction is used to estimate shaft resistance. Cone penetration causes significant soil deformation, which makes the CPTu results particularly suitable for assessing ultimate limit states of pile capacity. Building upon the assumption of CPT as a mini-pile, several methods have been developed in recent years across different countries, which can be applicable to pile design. Among modern approaches, one can find the LCPC method [2], Unicone, [7], ICP method [8], German method [9], UWA-05 [10], Togliani’s method [11], CPT 2012 [12], and SEU method [13].

Outside the CPTu-based methods, there are solutions that rely on pressuremeter testing (PMT) [14,15], mainly used in France and Canada where the method is popular, as well as emerging methods based on flat dilatometer testing (DMT) [16,17]. Both PMT and DMT soundings induce similar, moderate levels of soil, and are more sensitive to stress changes in the subsoil. Therefore, results of these tests are more suitable for deformation analysis.

The mobilization of shaft and base resistance in piles is often described using the transfer functions method. Transfer functions for axial loading were initially developed more than 70 years ago [18]. Since that time, various methods have been proposed to address specific pile types and soil conditions. These curves differ in form, complexity, and the number of parameters required. Examples include non-linear functions [19], trilinear functions [20,21], hyperbolic curves [22,23], visco–elastic models [24], as well as root curves [25]. Current approaches mainly rely on results from CPT/CPTu, PMT soundings, or static load testing (SLT). Although DMT results are not commonly used in direct pile design, the measured parameters can be appropriate for estimating pile bearing capacity. Transfer functions utilizing DMT sounding results are still in the early stages of development. Preliminary attempts have been made to develop such transfer curves for CMC displacement columns, using data obtained from in-situ testing of soft soils in Northern Poland [26]. A simplified tri-linear curve was proposed, depending on the dilatometer modulus values. The maximum shaft resistance values were correlated with the pressure applied to the dilatometer membrane prior to the start of the sounding. An alternative approach was introduced for screw displacement piles (DPDT and SDP), where power functions were used to estimate ultimate shaft and base resistances [17].

During pile axial loading, the load is transferred from the structure to the soil through a contact zone commonly referred to as the interface, which is a thin shear zone formed around the pile, where shear stresses and pile displacements reach their maximum values. Beyond this shear zone, there is a transition zone in which shear strains gradually decrease with increasing distance from the pile shaft, eventually becoming negligible. The conditions occurring at the soil–pile interface can be successfully simulated using a direct shear interface test [27], in which one half of the shear box is filled with a structural material representative of the actual construction, while the other half is filled with the tested soil. The most accurate representation of real soil–structure interaction can be achieved by performing tests under constant normal stiffness (CNS) conditions [28]. However, depending on soil stiffness and soil type, CNS conditions can bias toward constant normal load condition (CNL). This is in particular true for soft soils.

**Figure 1:**
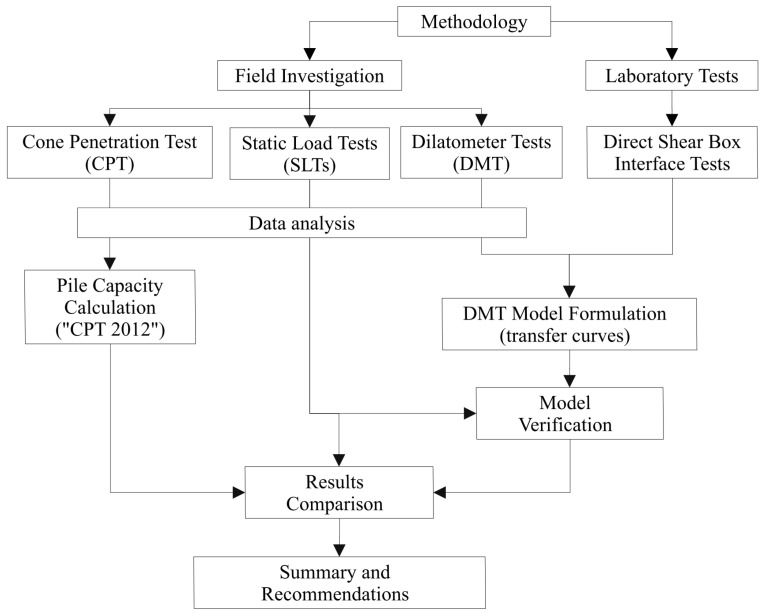
Flowchart summarizing the research methodology.

The aim of this article is to describe the local mobilization of shaft friction of compression and tension Controlled Modulus Columns, also referred as piles, using transfer functions based solely on DMT results, determined for forward and backward interface shearing, later also called reversal loading conditions. As a result, the novel DMT model, which incorporates reversal loadings and DMT soundings only, will be established to calculate the unit shaft friction of screw displacement piles in CMC technology. To achieve this goal, a series of laboratory direct shear tests were conducted on soil-concrete interfaces in both loading directions, where maximum interface shear strength was determined. Based on the laboratory results and DMT measurements, transfer functions were proposed that utilize the reversal loading, dilatometer modulus (*M_DMT_*), and the initial horizontal stress (*p*_0_) in DMT measurement. Subsequently, the obtained results were compared with those obtained from the ‘CPT 2012′ method presented in the French pile design standard AFNOR NF P94-262:2012-07 [29]. The methodology flowchart for described process is presented in Figure 1. It outlines the key stages of the methodology, described in detail in the following sections of this paper.

## 2. Geotechnical Site Investigation

Field investigations for this study were conducted in the Vistula Marshlands, Northern Poland, during construction of the S7 expressway. The testing site was located near the Nogat River, and due to the region’s geological history, the encountered soil conditions can be considered representative for the entire Vistula Marshlands. Comprehensive description of the testing site can be found in [30,31]. For the purpose of this research, only a brief review is given. The soil profile consists of alternating layers of soft soils of high compressibility (including organic silts, clays, and peat) and sandy deposits. Groundwater was encountered at approximately 1.7 m below ground level.

### 2.1. DMT Soundings

Eight DMT tests were carried out according to ASTM D6635 [32]. For the subsequent analysis, as well as for the design of the laboratory testing program, the vertical drained constrained modulus *M_DMT_* need to be determined [33] using formula:

*M_DMT_* = *R_M_* ∙ *E_D_* (1)

where: *R_M_* = correction factor [-], and *E_D_* = dilatometer modulus [kPa], along with the effective horizontal stress ${\sigma \prime }_{h}$, calculated as:

*σ’_h_* = *K* ∙ *σ’_v_* (2)

where: *σ’_v_* = effective vertical stress [kPa], *K* = lateral earth pressure coefficient.

The coefficient *K* was estimated using Marchetti’s equation [33] for cohesive soils:

*K* = (*K_D_*/1.5)^0.47^ − 0.6 (3)

where: *K_D_* = horizontal stress index [-].

For granular soils, the method proposed by Hossain and Andrus [34] was adopted:

*K* = 0.72 + 0.456 ∙ *log*(*OCR*) + 0.035 ∙ *K_D_* − 0.194 ∙ *log*(*q_c_*/*σ’_v_*) (4)

where: *q_c_* = cone resistance from CPTU [kPa], *OCR* = overconsolidation ratio [-], *K_D_* = horizontal stress index [-].

**Figure 2:**
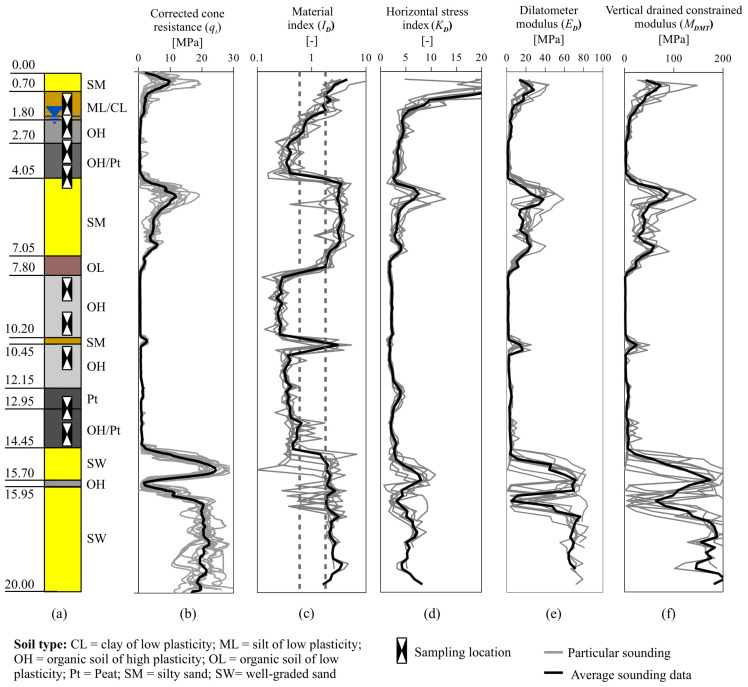
(**a**) Jazowa site soil profile, (**b**) corrected cone resistance; (**c**–**f**) DMT probing results.

Soil profile and DMT soundings results are shown in Figure 2. Particular soundings are shown with grey lines, while the black line represents the averaged profile. Sounding results are burden with low to moderate uncertainty. For parameters registered during soundings, the coefficient of variation was in the range of 10–20% for soft soils, while for sand deposits it was between 20% and 40%. Such a values are in agreement with reported soil variability [35]. Consequently, the test results are consistent, indicating a regular arrangement of the soil layers.

For each soil layer, the values of individual parameters from both CPT and DMT tests were averaged. Additionally, vertical drained constrained modulus *M_DMT_* and the effective horizontal stress *σ’_h_* were calculated. Six weeks after pile installation, additional DMT soundings were conducted near the shaft of one of the columns. Based on those soundings, *K_D_* value for the pile shaft was estimated, and effective horizontal stress after pile installation was calculated [36]. All these data are provided in Appendix A—Table A1.

### 2.2. Static Load Tests (SLT)

**Figure 3:**
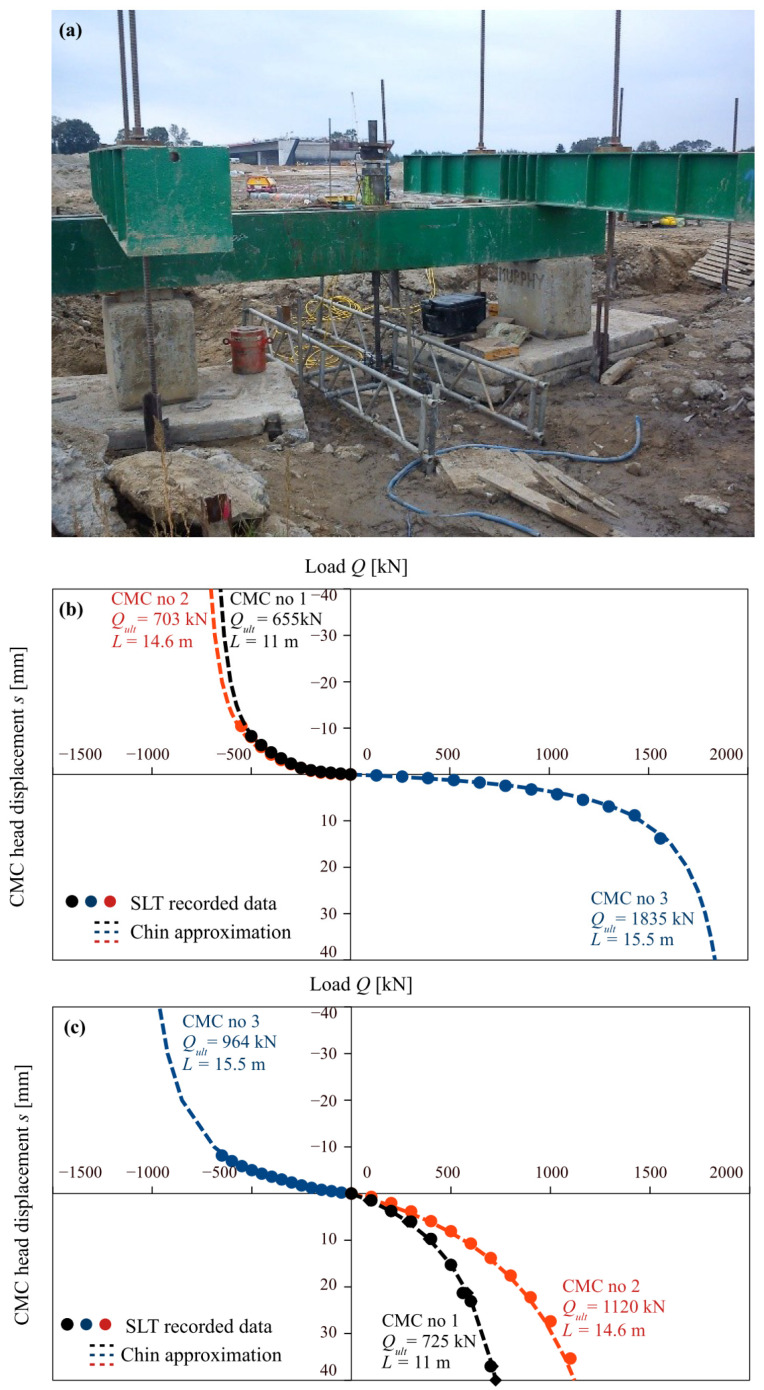
(**a**) static load test stand, (**b**) SLT results—first loading step; (**c**) SLT results—second loading step.

The piles were constructed in the form of concrete screw displacement piles using the CMC technology patented by Menard [37]. The piles varied in length, depending on the local soil profile. Three types of piles were distinguished: (1) floating pile (11 m long), embedded entirely in soft soil layers, without reaching bearing soils, (2) end-bearing pile (14.6 m long), resting directly on load-bearing layer, (3) embedded pile (15.5 m long), partially embedded in the load-bearing layer. The piles were tested under compression–tension and tension–compression schemes. The static load test stand is presented in Figure 3a. The tests aimed to estimate the tension capacity and assess the influence of prior tension or compression loading on subsequent pile performance.

The CMCs selected for analysis exhibited significant displacements during static load tests, which facilitates analysis of pile response and determination of ultimate pile bearing capacity. The results of the load tests for these columns are presented in Figure 3. The 11 m (CMC no 1) and 14.6 m (CMC no 2) columns were first subjected to tension (uplift) loading and then to compression loading, whereas the longest column, 15.5 m (CMC no 3) in length, was first loaded in compression and subsequently in tension. The ultimate bearing capacity was assumed to be 10% of pile diameter and the load-settlement curves were extrapolated behind the range of field data using Chin’s method [38]. As one can see, first loading direction is characterized by a much stiffer response. This is very characteristic behavior of soils subjected to alternating loads, as presented in many previous studies of soils [39] and pile loadings [40].

## 3. Lab Soil–Concrete Interface Tests

### 3.1. Testing Procedure

Firstly, the stiffness at the soil–pile interface was estimated. Dilatometer test results were utilized to calculate the shear modulus [41], and subsequently the stiffness of the surrounding soil:

*k* = 4*G*/*d* (5)

where: *G* = shear modulus of soil [kPa], *d* = pile diameter [mm].

In cohesive soils, the estimated stiffness ranged from 4 to 44 kN/mm. These low values are considered negligible and were assumed to be zero from a practical point of view, resulting in CNL conditions. For grained soils, higher stiffness values were obtained––approximately 200 kPa/mm for the upper sand layer and over 300 kPa/mm for sands located below a depth of 16 m. Since the tested piles were not embedded in these dense sand layers, and the influence of the pile base seems to be limited, adopting Constant Normal Load (CNL) conditions (i.e., *k* = 0) was deemed a reasonable simplification. Two types of soil-concrete interfaces, smooth and rough concrete, were examined, using soil samples from the test site. A detailed scheme of the interface preparation as well as the soil specimen preparation procedures is presented by Konkol and Mikina [27]. All samples were tested according to ASTM D3080 [42] in 60 × 60 mm shear box. The shear rate was adjusted to reflect the average pile push-in/pull-out rate observed during the static load tests, i.e., 0.06 mm/min. Normal stress levels applied to the samples during interface testing corresponded to the normal stress acting on the pile shaft in each respective soil layer. Each sample was sheared in reversal load conditions, and the procedure was repeated for both smooth and rough concrete interfaces.

### 3.2. Interface Tests Results

#### 3.2.1. Fine-Grained Soils

**Figure 4:**
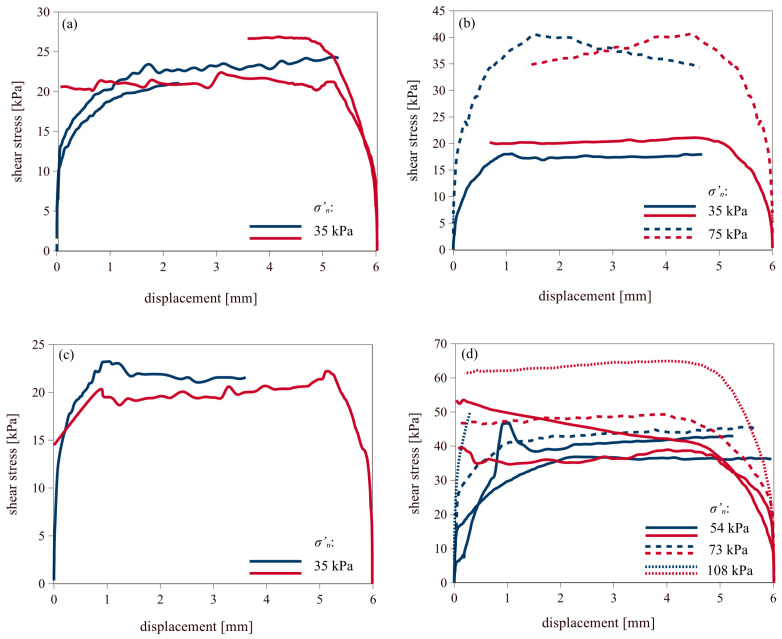
Results of forward (blue line) and backward (red line) shearing of fine soils on smooth concrete: (**a**) silt/clay; (**b**) peat; (**c**) organic clay; (**d**) organic silt.

**Figure 5:**
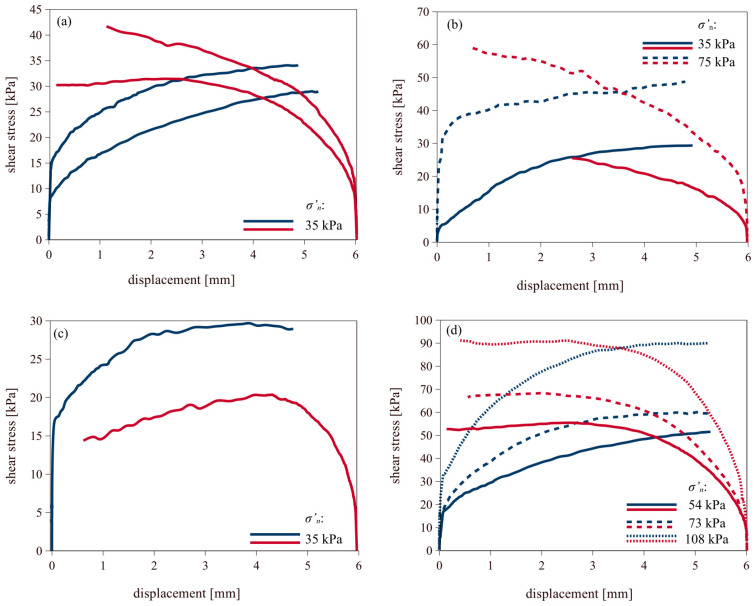
Results of forward (blue line) and backward (red line) shearing of fine soils on rough concrete: (**a**) silt/clay; (**b**) peat; (**c**) organic clay; (**d**) organic silt.

Figure 4 and Figure 5 present the shear test results of fine-grained soils against smooth and rough concrete, respectively. Shearing in the forward direction is shown in blue, while backward shearing is shown in red. For the smooth concrete interface, the maximum shear strength was reached at displacements of approximately 1 mm, whereas for the rough concrete interface, peak values were observed at displacements around 2 mm or at even higher values. The plots clearly demonstrate an increase in shear stress with increasing normal stress applied during the tests. Additionally, a slight increase in maximum shear strength can be observed during backward shearing. In those cases, peak values were reached at displacements of about 1 mm for the smooth concrete interface, and slightly above 1 mm for the rough concrete interface.

#### 3.2.2. Coarse-Grained Soils

**Figure 6:**
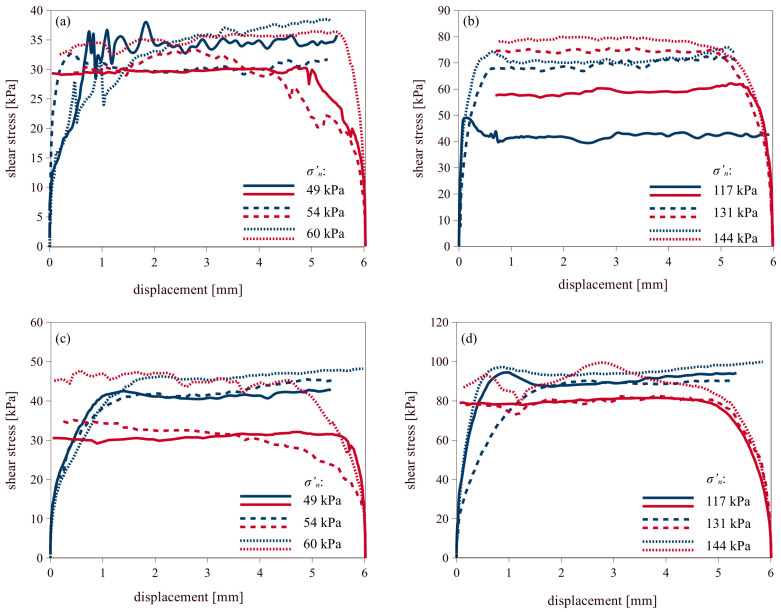
Results of forward (blue line) and backward (red line) shearing of coarse-grained soils on smooth concrete: (**a**) medium dense sand; (**b**) dense sand, and on rough concrete: (**c**) medium dense sand; (**d**) dense sand.

Figure 6 presents the results of shear tests on coarse-grained soils in both directions. As in the previous figures, forward shearing is shown with red lines, and backward shearing with blue lines. The graphs show an increase in shear stress with increasing normal stress, both for medium-dense and dense sands. Maximum shear stresses were mobilized at displacements between 1–2 mm for medium sands and at lower displacement, at approximately 0.5–1 mm, for dense sands. For medium sands, the results of backward shearing were similar to those of forward shearing. However, in dense sands, an increase in shear stress could be observed during reverse shearing on smooth interface.

## 4. Formulation of DMT Model

### 4.1. Basic Idea

**Figure 7:**
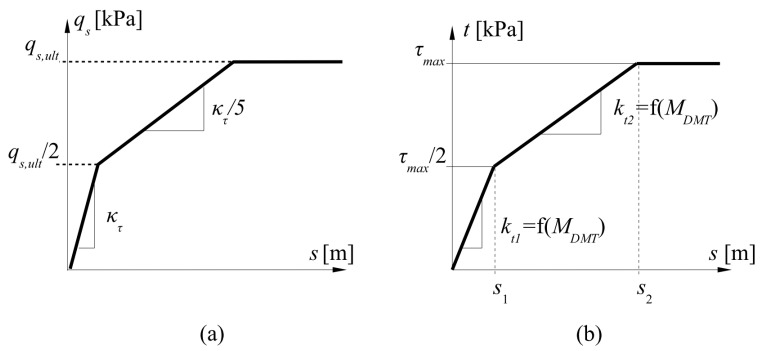
(**a**) The shape of Frank and Zhao [12] t–z curve for shaft friction; (**b**) The concept of DMT model for unit shaft friction. Note: *q_s_* = shaft unit resistance; *q_s,ult_* = ultimate shaft unit resistance; *κ_t_* = slope parameter; *s* = pile displacement; *τ_max_* = maximum interface shear strength; *k_t_*_1_*, k_t_*_2_ = slope parameters.

The method proposed in this article builds upon the well-known transfer functions introduced by Frank and Zhao [12,14,15], which utilize results from the PMT (Figure 7a). The three-linear functions are compromises between scientific quality and engineering applicability, where ease of use is a primary requirement. Based on this concept, a corresponding relationship was developed using the *M_DMT_* instead of the pressuremeter modulus (Figure 7b). The characteristics required to describe this model were defined based on results from direct shear interface tests.

### 4.2. DMT Model

From each direct shear test, the curve corresponding to the normal stress acting on the pile shaft after installation (calculated at the midpoint of each soil layer) was selected. This curve was then described according to the scheme presented in Figure 7b, yielding the slopes *k_t_*_1_ and *k_t_*_2_ at the given normal stress. Additionally, considering all test results for a given soil, the relationships between maximum shear stress *τ_max_* for samples sheared on smooth and rough interfaces in reversal loading were determined. After analyzing all the graphs, a summary of all parameters for tests on smooth concrete (Table A2) and rough concrete (Table A3) was prepared. These tables include: depth ranges of soil layers, type of soil, averaged dilatometer modulus (before pile installation), horizontal stress acting in the middle of a given soil layer (before pile installation), coefficients *k_t_*_1_ and *k_t_*_2_*, τ_max_* calculated for a given normal stress using relations *τ_max_* -*σ_n_*.

Based on the data in Table A2 and Table A3, relationships were established between the coefficients *k_t_*_1_ and *k_t_*_2_ and the dilatometer modulus *M_DMT_*, separately for tests conducted on smooth and rough concrete surfaces, and for shearing in both directions. The full derivation of these functions can be found in [36]. Plots based on linear, polynomial, logarithmic, power, and exponential regression models were tested. The best-fit function was calculated using the least squares method. After analyzing all the plots, the power regression model was selected, as it exhibited the highest coefficient of determination (*R*^2^). The *R*^2^ was calculated as *R*^2^ =1 − *S_res_*/*S_tot_* where *S_res_* = residual sum of squares, and *R_tot_* = sum of total squares. For cohesive soils on smooth concrete, the coefficients of determination were 0.9353 and approximately 0.70 for *k_t_*_1_ and *k_t_*_2_, respectively, and 0.6526 and 0.8732 for rough concrete. In the case of coarse-grained soils, the values were approximately 0.79 for smooth concrete and 0.80 for rough concrete. The equations representing the relationships between the parameters *k_t_*_1_, *k_t_*_2_ and *M_DMT_* are presented in the Table 1.

**Figure 8:**
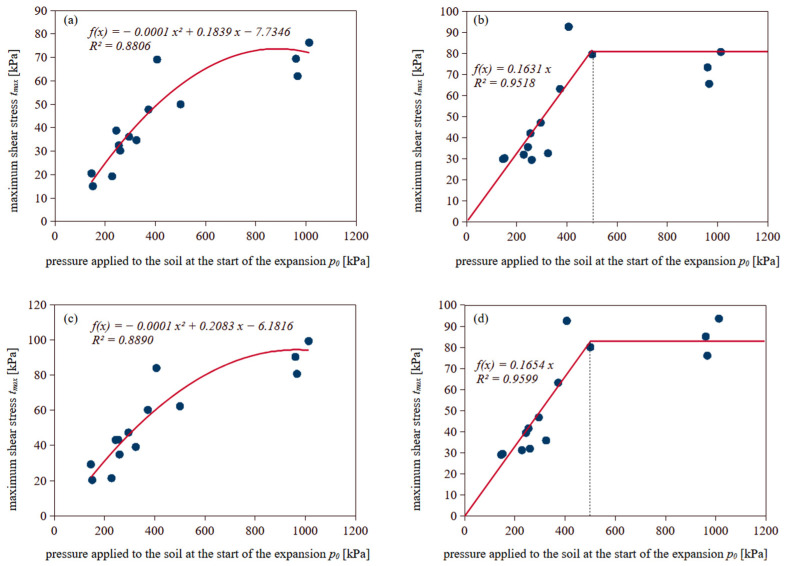
Relationship between *p*_0_ and *τ_max_* for: (**a**) smooth interface—forward shearing; (**b**) smooth interface—backward shearing; (**c**) rough interface—forward shearing; (**d**) rough interface—backward shearing.

Construction of the t–z curve (Figure 7b) requires knowledge of the maximum shear stress mobilized at the interface within each soil layer. To avoid performing direct shear tests for every case, relationships were developed that allow for the estimation of *τ_max_* based on parameters obtained from dilatometer tests. Specifically, previously determined values of *τ_max_* for both smooth and rough concrete interfaces were correlated with parameters measured during DMT soundings. The strongest correlation was observed with *p*_0_, the pressure recorded at the beginning of membrane expansion. This value was averaged within each soil layer using data from all pre-installation DMTs. An attempt was made to identify the highest possible correlation between the maximum shear stress and *p*_0_. The coefficient of determination (*R*^2^) was used as an indicator. Figure 8 shows the relationships between the maximum shear stress *τ_max_* derived from the transformation functions and the initial expansion pressure *p*_0_ measured in the DMT.

## 5. Model Verification and Discussion

### 5.1. Pile Bearing Capacity According to DMT Model

In accordance with the methodology presented in the previous sections, the maximum shaft friction resistance during pile compression and tension was estimated. For selected piles, the results of the nearest DMT soundings were analyzed to determine the vertical drained constrained modulus. The obtained relationships used in the calculations are presented in Figure 8. Subsequently, the coefficients *k_t_*_1_ and *k_t_*_2_ were determined, which allowed the construction of transformation curves for each probing depth, with a step of 0.20 m. Assuming simplified conditions such as constant pile shape and diameter along the entire length, homogeneous soil, constant modulus, and a linearly decreasing applied force with depth, displacements were estimated for successive load steps at all calculation depths. Based on these, the shaft friction values at specific depths under given loads were determined. By multiplying these values by the pile shaft surface area and summing along the pile length, the total shaft bearing capacity for each load step was obtained. The calculations were repeated for subsequent loads, resulting in *Q*–*s* curves for each pile.

**Figure 9:**
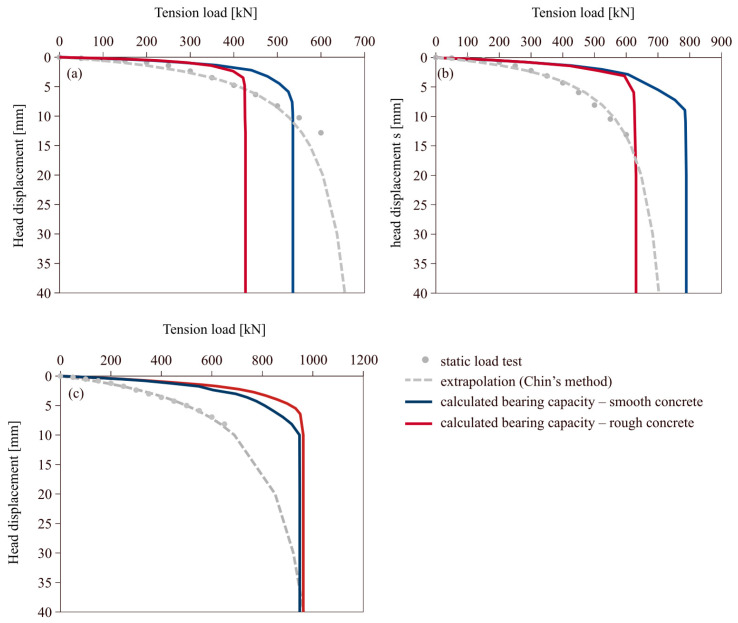
Results for the: (**a**) CMC 1 (11-m column); (**b**) CMC 2 (14.6-m column); (**c**) CMC 3 (15.5-m column).

Three piles were analyzed: 11 m, 14.6 m, and 15.5 m long. The first two piles were initially pulled out and then pushed in, whereas the longest pile was first pushed in and then pulled out. The results of the shaft bearing capacity calculations during tension load for these piles are presented in Figure 9, applying the relations for forward shearing for the shorter piles, and the relations for backward shearing for the longest pile.

### 5.2. DMT Model Versus CPT 2012

The CPT 2012 [29] method is one of the newer methods for direct pile design. Using this method, the shaft capacity of selected piles was estimated for later comparison with the proposed calculation model. The CPT 2012 method is fully compatible with the guidelines of Eurocode 7, and its basic assumption is to use only the cone resistance *q_c_* (or corrected cone resistance *q_t_*) in the calculations. The calculated shaft capacity is as follows:

*R_t_* = *Σq_si_* ∙ *A_si_* (6)

where: *q_si_* = pile shaft resistance corresponding to i layer, and *A_si_* = pile shaft area corresponding to the i layer.

According to the pile classification proposed in the AFNOR standard [29], CMC columns can be classified as Class C3 and Category 7. In order to determine the soil-type parameters required for the calculations, it was necessary to convert the current classification into the European Soil Classification System (ESCS) used in the French standard. A detailed calculation procedure and the results of the total bearing capacity for the 11 m and 14.6 m columns were presented in an earlier publication [43]. Analogous calculations were carried out for the 15.5 m column, and the results of shaft capacity are summarized in Table 2. In the case of calculation values in the standard, no distinction is made between whether the column is subjected to compression or in tension. This aspect is only taken into account at the design stage by applying appropriate reduction factors [43].

Analyzing the obtained results, it can be observed that the shaft capacities calculated for rough concrete during shearing in the first direction are approximately 20% higher than those for smooth concrete. At the same time, it is evident that, for calculations in the opposite direction, the results are practically independent of the type of concrete used. However, an increase in shaft capacity during reverse shearing is visible––approximately 40% for smooth concrete and shorter columns, and around 14% for rough concrete and shorter columns. For the longest column, an increase is observed only in the case of smooth concrete, while the results for rough concrete remain similar. When comparing the shaft results obtained with the proposed method to those from the CPT 2012 method, it can be concluded that the uplift capacities for the 11 m and 14.6 m columns are lower than the compression capacities. Furthermore, the results obtained for rough concrete are closer to those from the CPT 2012 method (with differences ranging from 7% to 30%). For the 15.5 m column, the calculated values (except for the case of smooth concrete under compression) differ by only about 7% from those given by the CPT 2012 method, indicating a reasonably good agreement. All methods also demonstrate very good concurrence with SLT results indicating their applicability.

### 5.3. Discussion on Overall DMT-Model Performance

A key novelty of this study is the introduction of a method that uses solely DMT soundings to derive both ultimate bearing capacity and the load–displacement curves for CMCs. This contrasts with most traditional methods requiring multiple test types or empirical adjustments. The DMT method’s ability to deliver reliable predictions using only one in situ testing method (DMT) is highly advantageous for routine design in regions like Poland, where DMT is already widespread and produces dense data profiles (every 20 cm), avoiding the need for subjective averaging by soil layers. The method has been developed and calibrated for screw auger piles in CMC technology, and its current form does not account for pile group effects. Caution is advised when applying the model to:

- Driven or pushed-in piles, where high residual stress or grain crushing at the interface may alter behavior not replicable in shear box tests.
- Very long piles, where friction fatigue may reduce shaft resistance along the upper segments.
- Pile base zones, where high stress concentration may lead to friction mobilization that cannot be accurately modeled by interface shear tests.

The simplified model of the transfer functions does not precisely fit the shape of the *Q*–*s* curves. This observation is based purely on engineering judgment. Firstly, the direct shear boxes produce too stiff a response in the initial part of the interface shearing. Application of simple shear mode could improve the results. Secondly, the assumption of marginal influence of the pile base on the shaft response to tension and compression shaft friction can be inaccurate. There is evidence that loading direction influences the magnitude of shaft unit resistance under tension and compression [44]. The CNS test for sand–concrete interface is a possible solution for such a scheme, especially for compression loading. However, the proper stiffness can be only determined when instrumented load tests will be performed and unit shaft friction directly measured. Finally, using a different type of transfer function could result in an overall better fit.

Thus, to improve and generalize the DMT method, the following recommendations are given:

- Interface shear testing under CNS conditions, particularly in medium to dense sands and stiff clays, to better capture realistic interface stress paths.
- Increase allowable displacements in shear tests (beyond 4 mm), especially for soft soils.
- Apply and calibrate the method across a broader range of pile types and soil conditions to validate its universal applicability and incorporate installation effects into routine pile design frameworks.

## 6. Conclusions

The present research represents the first attempt to establish relationships between parameters obtained from Dilatometer Test (DMT) soundings and the skin friction of CMC of varying lengths and schemes of loading. The presented results allow the following conclusions to be drawn:

- The obtained results for the concrete–soil interface show a closer agreement with the skin friction capacity derived from methods based on Cone Penetration Test (CPT) data (“CPT 2012”).
- Laboratory direct shear interface tests conducted in both forward and backward directions highlighted the importance of considering loading reversals to accurately capture the behavior of soil-concrete interfaces.
- The DMT-based transfer curves provide an improved and practical tool for modeling pile–soil interaction, applicable to a wide range of soil types and pile installation scenarios encountered in the field.

## Figures and Tables

**Table 1:** The slopes coefficients of the t–z curves as a function of DMT modulus for smooth and rough concrete interface in reversal loading.

		Fine-Grained Soils	Coarse-Grained Soils
Smooth interface	Forward shearing	*k_t_*_1_ = 0.7553 · (*M_DMT_*)^0.5825^	*k_t_*_1_ = 0.0091 · (*M_DMT_*)^0.8747^
		*k_t_*_2_ = 0.1051 · (*M_DMT_*)^0.6390^	*k_t_*_2_ = 0.0003 · (*M_DMT_*)^1.0156^
	Backward shearing	*k_t_*_1_ = 2.0073 · (*M_DMT_*)^0.3976^	*k_t_*_1_ = 0.1126 · (*M_DMT_*)^0.6735^
		*k_t_*_2_ = 0.0289 · (*M_DMT_*)^0.6806^	*k_t_*_2_ = 0.0641 · (*M_DMT_*)^0.5610^
Rough interface	Forward shearing	*k_t_*_1_ = 0.0248 · (*M_DMT_*)^0.9558^	*k_t_*_1_ = 0.1946 · (*M_DMT_*)^0.5671^
		*k_t_*_2_ = 0.1098 · (*M_DMT_*)^0.5348^	*k_t_*_2_ = 0.0033 · (*M_DMT_*)^0.7950^
	Backward shearing	*k_t_*_1_ = 3.0346 · (*M_DMT_*)^0.3896^	*k_t_*_1_ = 3.0515 · (*M_DMT_*)^0.3339^
		*k_t_*_2_ = 0.0248 · (*M_DMT_*)^0.7458^	*k_t_*_2_ = 0.0133 · (*M_DMT_*)^0.6826^

**Table 2:** DMT-model versus “CPT 2012”.

Pile Length [m]	Load Scheme	Shaft Resistance Form SLT [kN]		Shaft Resistance in Tension Load [kN]		Shaft Resistance in Compression Load [kN]		CPT 2012
		Tension	Compression	Smooth Interface	Rough Interface	Smooth Interface	Rough Interface	
11	T-C	655	671	426.81	535.65	603.37	611.57	688
14.6	T-C	703	949	630.85	789.27	887.56	899.68	841
15.5	C-T	964	1215	948.26	962.26	769.14	970.03	1018

## Data Availability

The original contributions presented in this study are included in the article. Further inquiries can be directed to the corresponding author.

## Appendix A

Averaged results of cone penetration tests and flat dilatometer tests are presented in Table A1.

**Table A1:** Averaged results of cone penetration tests and flat dilatometer tests.

Before Installation								After Installation	
Soil Layer	*q_t_* AVG	*I_D_* AVG	*K_D_* AVG	*E_D_* AVG	*M_DMT_* AVG	*K*	*σ′_h_*	*K*	*σ′_h_*
	[MPa]	[-]	[-]	[MPa]	[MPa]	[-]	[kPa]	[-]	[kPa]
SM	6.85	-	-	-	-	-	-	-	-
ML/CL	1.99	1.84	10.51	13.32	15.6	1.80	36.47	1.80	36.47
OH	0.54	0.82	4.42	3.90	6.0	1.06	34.45	1.06	34.45
Pt/OH	0.51	0.38	3.47	1.74	2.3	0.88	33.56	0.88	33.56
SM	6.00	2.93	4.18	21.93	67.9	0.64	31.25	1.09	53.46
					31.8	0.52	32.05	0.96	59.13
OL	1.37	1.58	1.79	8.00	-	0.48	35.08	0.48	35.08
OH	0.55	0.26	2.17	1.601	1.5	0.59	48.39	0.59	48.39
SM	2.22	2.02	1.84	11.74	17.8	0.50	45.00	0.53	47.52
OH	0.83	0.35	2.09	2.46	2.1	0.57	54.32	0.57	54.32
Pt	1.07	0.37	3.59	4.73	7.7	0.90	92.47	0.90	92.47
OH/Pt	1.05	0.49	2.54	4.34	3.8	0.67	72.92	0.67	72.92
SW	1, 5.46	2.14	6.38	56.65	140.1	0.73	83.06	1.02	117.14
OH	2.57	1.88	3.86	35.61	5.7	0.89	107.91	0.89	107.91
SW	17.37	2.51	5.84	69.22	137.4	0.70	90.34	1.02	131.08
					148.1	0.64	91.29	1.02	144.46

Note: *q_t_* = corrected cone resistance, *I_D_* = material index; *K_D_* = horizontal stress index; *E_D_* = dilatometer modulus, *M_DMT_* = drained constrained modulus; *K* = lateral earth pressure coefficient, *σ′_h_* = lateral effective stress, AVG = denotes average value along the soil layer.

## Appendix B

Soil and DMT model parameters for soil—concrete interface.

**Table A2:** Comparison of the relationships between the slope of the t–z curve and the DMT modulus for smooth concrete interface.

Depth of Soil Layer [m]		Soil Type	*M_DMT_*	*σ_n_*	Forward Shearing			Backward Shearing		
					*τ_max_*	*k_t_*_1_	*k_t_*_2_	*τ_max_*	*k_t_*_1_	*k_t_*_2_
Top	Bottom		[kPa]	[kPa]	[kPa]	[-]	[-]	[kPa]	[-]	[-]
0.00	0.70	SM	90,056	18.0	-	-	-	-	-	-
0.70	1.80	ML/CL	15,556	36.0	19.28	192.80	8.39	23.44	78.1	5.4
1.80	2.70	OH	5960	34.0	15.05	150.50	6.55	22.40	74.6	5.2
2.70	4.05	Pt/OH	2290	33.5	20.52	73.26	10.67	22.14	37.0	3.0
4.05	5.55	SM	67,857	53.5	28.48	71.19	10.96	34.34	114.3	20.2
5.55	7.05	SM	31,791	59.0	31.50	78.75	12.13	37.41	124.6	22.0
7.05	7.80	OL	-	35.0	-	-	-	-	-	-
7.80	10.20	OH	1541	48.0	32.50	54.28	13.55	29.69	37.1	5.7
10.20	10.45	SM	17,819	47.5	25.18	62.94	9.69	30.99	103.2	18.2
10.45	12.15	OH	2100	54.0	36.16	60.39	15.08	32.81	41.0	6.3
12.15	12.95	Pt	7667	92.5	50.02	178.57	26.01	52.85	88.3	7.1
12.95	14.45	Pt/OH	3757	73.0	47.75	79.74	19.91	42.70	53.4	8.2
14.45	15.70	SW	140,054	117.0	62.01	310.05	62.01	69.80	349.0	49.8
15.70	15.95	OH	5688	108.0	69.10	115.40	28.81	60.92	76.1	11.7
15.95	17.50	SW	137,412	131.0	69.43	347.15	69.43	77.62	388.1	55.4
17.50	19.00	SW	148,054	144.0	76.32	381.60	76.32	84.88	424.4	60.6

Note: *σ_n_* = normal stress; *M_DMT_* = drained constrained modulus; *τ_max_* = maximum interface shear strength; *k_t_*_1_*, k_t_*_2_ = slope parameters.

**Table A3:** Comparison of the relationships between the slope of the t–z curve and the DMT modulus for rough concrete interface.

Depth of Soil Layer [m]		Soil Type	*M_DMT_* AVG	*σ_n_* AVG	Forward Shearing			Backward Shearing		
					*τ_max_*	*k_t_*_1_	*k_t_*_2_	*τ_max_*	*k_t_*_1_	*k_t_*_2_
Top	Bottom	[-]	[kPa]	[kPa]	[kPa]	[-]	[-]	[kPa]	[-]	[-]
0.00	0.70	SM	90,056	18.0	-	-	-	-	-	-
0.70	1.80	ML/CL	15,556	36.0	21.42	214.20	3.64	31.95	106.4	7.4
1.80	2.70	OH	5960	34.0	20.33	203.30	3.46	30.27	100.8	7.0
2.70	4.05	Pt/OH	2290	33.5	29.27	14.64	5.85	29.85	49.8	4.0
4.05	5.55	SM	67,857	53.5	39.18	65.43	11.36	37.80	94.5	14.6
5.55	7.05	SM	31,791	59.0	43.14	72.04	12.51	41.21	103.0	15.9
7.05	7.80	OL	-	35.0	-	-	-	-	-	-
7.80	10.20	OH	1541	48.0	43.24	43.24	6.18	42.05	52.6	8.1
10.20	10.45	SM	17,819	47.5	34.86	58.22	10.11	34.09	85.2	13.1
10.45	12.15	OH	2100	54.0	47.32	47.32	6.76	47.10	58.9	9.0
12.15	12.95	Pt	7667	92.5	62.31	31.16	12.46	79.51	132.8	10.7
12.95	14.45	Pt/OH	3757	73.0	60.24	60.24	8.61	63.10	78.9	12.1
14.45	15.70	SW	140,054	117.0	80.73	161.46	42.79	76.05	152.1	44.7
15.70	15.95	OH	5688	108.0	84.04	84.04	12.01	92.56	115.7	17.8
15.95	17.50	SW	137,412	131.0	90.39	180.78	47.91	85.15	170.3	50.1
17.50	19.00	SW	148,054	144.0	99.36	198.72	52.66	93.60	187.2	55.0

Note: *σ_n_* = normal stress; *M_DMT_* = drained constrained modulus; *τ_max_* = maximum interface shear strength; *k_t_*_1_*, k_t_*_2_ = slope parameters.
